# NORAD modulates miR-30c-5p-LDHA to protect lung endothelial cells damage

**DOI:** 10.1515/med-2022-0446

**Published:** 2022-04-06

**Authors:** Yuhua Zhou, Chunyan Chen, Qingtian Li, Huiqiu Sheng, Xiaokui Guo, Enqiang Mao

**Affiliations:** Department of Emergency, Ruijin Hospital, Shanghai Jiao Tong University School of Medicine, Shanghai, 200025, China; Key Laboratory of Parasite and Vector Biology, Ministry of Health, Chinese Center for Tropical Disease Research, Shanghai Jiao Tong University School of Medicine, Shanghai, 200025, China; Department of Laboratory Medicine, Ruijin Hospital, Shanghai Jiao Tong University School of Medicine, Shanghai, 200025, China

**Keywords:** lncRNA NORAD, acute lung injury, HPMECs, miR-30c-5p, glucose metabolism, LDHA

## Abstract

Acute lung injury (ALI) is a devastating human malignancy characterized by excessively uncontrolled inflammation and lung endothelial dysfunction. Non-coding RNAs play essential roles in endothelial protections during the pathological processes of ALI. The precise functions and molecular mechanisms of the lncRNA-NORAD-mediated endothelial protection remain obscure. This study reports NORAD was significantly induced in human pulmonary microvascular endothelial cells (HPMECs) under lipopolysaccharide (LPS) treatment. Silencing NORAD effectively protected HPMECs against the LPS-induced cell dysfunction. In addition, RNA pull-down and luciferase assay validated that NORAD sponged miR-30c-5p, which showed reverse functions of NORAD in the LPS-induced cell injury of HPMECs. Furthermore, the glucose metabolism of HPMECs was significantly elevated under LPS stimulation which promoted the glucose consumption and extracellular acidification rate (ECAR) of HPMECs. Inhibiting NORAD or overexpressing miR-30c-5p suppressed glucose metabolism in HPMECs, leading to protective effects on HPMECs under LPS stimulation. The glycolysis key enzyme, lactate dehydrogenase-A (LDHA), was subsequently identified as a direct target of miR-30c-5p. Finally, recovery of miR-30c-5p in NORAD-overexpressing HPMECs effectively overrode the NORAD-promoted glycolysis and impaired endothelial dysfunction under LPS stimulation by targeting LDHA. Summarily, we demonstrated a NORAD-miR-30c-5p-LDHA-glycolysis axis in the LPS-induced HPMECs dysfunction *in vitro* and *in vivo*, contributing to the development of anti-ALI therapeutic approaches.

## 1 Introduction

Acute lung injury (ALI) is a devastating human malignancy with high incidence and mortality [1]. ALI is characterized by excessive uncontrolled inflammation, lung endothelial dysfunction, and oxidative damage, leading to progress towards acute respiratory distress syndrome (ARDS) [2]. Thus, it is urgent to investigate effectively the therapeutic approaches for ALI treatment. Accumulating evidence revealed that pulmonary endothelium plays essential roles and acts as an orchestrator of ALI [3]. In the lining of pulmonary vessel, endothelial cells (ECs) are tightly connected to adjacent ones through intercellular junctions, forming a semipermeable barrier which functions in lung homoeostasis via regulating the transport of fluids, proteins, and inflammatory factors [4,5]. In response to stimuli, such as lipopolysaccharide (LPS), reactive oxygen species, or other pathological factors, barrier disruption occurs in ECs and proinflammatory proteins are secreted by injured ECs [6]. Therefore, targeting the dysregulated endothelial induced by stimuli will be a potentially effective strategy for ALI treatment.

Long non-codingRNAs (lncRNAs) are a family of RNAs with relatively larger size (longer than 200 nucleotides), and non-protein coding capacity [7]. They function as scaffolds or decoys of miRNAs or mRNAs in cytoplasm or nucleus via regulating transcriptional or posttranscriptional processes and epigenetic modifications [8]. Studies have revealed that lncRNAs are important mediators in various cellular processes including proliferation, migration, stress response, metabolism, inflammation, and cell death [9]. Moreover, research works have indicated fundamental roles of lncRNAs in acute lung inflammation and infectious diseases [10]. A recent study uncovered that lncRNA MALAT1 participated in pulmonary pathogeneses via regulating the aberrant macrophage activation [11]. MALAT1 was significantly upregulated in LPS-treated macrophages and silencing MALAT1 effectively attenuates pro-inflammatory activation of aberrant macrophages [11], suggesting targeting the lncRNA-mediated molecular pathway may contribute to treatment of ALI. LncRNA activated by DNA damage (NORAD) has been reported to be significantly upregulated in diverse human cancers and contributes to lung cancer progressions, including proliferation [12], apoptosis [12], epithelial to mesenchymal transition [13], chemoresistance [14], and migration [15]. Currently, the precise roles and molecular targets of NORAD in the LPS-stimulated dysfunction of human pulmonary microvascular endothelial cells (HPMECs) as well as ALI remain unclear.

Studies have unveiled that lncRNAs sponge target miRNAs to form ceRNA networks, leading to sequestering miRNAs [8]. Currently, the underlying molecular targets of the NORAD-regulated HPMECs dysfunction under LPS stimulation remain unclear. This study aimed to investigate the roles of NORAD in the LPS-stimulated HPMECs dysfunction. The NORAD-participated ceRNA network as well as downstream targets of miRNA will be identified. Our results will present new insights for the non-coding RNA-mediated HPMECs dysfunction, providing a potentially therapeutic approach against ALI.

## 2 Materials and methods

### 2.1 ALI patient samples collection and HPMECs isolation

Ethics of this study was approved by the Ethics Committee of Ruijin Hospital, Shanghai Jiao Tong University School of Medicine and followed the ethical principles for medical research involving human subjects outlined in the Declaration of Helsinki. Surgeries of ALI patients were carried out in Ruijin Hospital, Shanghai Jiao Tong University School of Medicine between April 1, 2018 and July 20, 2019. ALI was diagnosed based on the regular diagnostic criteria for acute lung injury. Isolation of primary HPMECs was performed according to previous reports [16]. Written informed consent was obtained from all the participants.

### 2.2 Cell culture

HPMECs and A549 cells were obtained from ScienCell (San Diego, CA, USA) and cultured with Dulbecco’s modified Eagle medium (DMEM; Gibco, Carlsbad, CA, USA) which contains 10% fetal bovine serum (Gibco, Carlsbad, CA, USA) and 1% penicillin/streptomycin (Invitrogen, Carlsbad, CA, USA), and incubated at 37°C with 5% CO_2_. LPS (Sigma-Aldrich, Shanghai, China) was dissolved in phosphate-buffered solution (PBS) and the stock solution (5 mg/mL) was stored at −20°C. HPMECs were treated with LPS at a concentration of 10 μg/mL for 24 h. Oxamate was purchased from Sigma-Aldrich (Shanghai, China). Rabbit monoclonal anti-lactate dehydrogenase-A (LDHA) (#3582), Hexokinase 2 (HK2) (#2106), and β-actin (#4970) were purchased from Cell Signaling Technology (Danvers, MA, USA).

### 2.3 Cell transfection of miRNAs, siRNAs, and plasmids

HPMECs (1 × 10^5^ cells/well) were seeded in a 6-well plate and incubated for 24 h. siRNA, miRNA, plasmid DNA as well as their negative controls were transfected into cells using Lipofectamine 2000 reagent (Invitrogen, Carlsbad, CA, USA). miR-30c-5p precursor and NORAD siRNA were synthesized from GenePharma (Shanghai, China). miR-30c-5p precursor was transfected at 12.5 nM, and siRNA was transfected at 50 nM. Plasmid DNA was transfected at 2 µg/mL. 48 h post transfection, the cells were collected and subjected to downstream experiments. Experiments were performed in triplicate.

### 2.4 Prediction of the lncRNA-miRNA and miRNA-mRNA interactions

The binding sites of miR-30c-5p on NORAD and LDHA 3′UTR were predicted from starBase 2.0 according to previous descriptions [17].

### 2.5 RNA isolation and qRT-PCR

Total RNA was isolated from cells using Trizol (Sigma, Shanghai, China) according to the manufacturer’s instructions. The concentration and quality of RNA samples were measured with a spectrophotometer NanoDrop 2000 (Thermo Fisher, Waltham, MA, USA). Complementary DNA was synthesized using 1 μg RNA with a PrimeScript™ RT reagent Kit (Takara). RT-qPCR reactions were performed using a Real-time PCR Mixture assays kit (TaKaRa, Dalian, China) on a CFX96 Real-Time PCR System (Applied Biosystems, Foster City, CA, USA). The primer sequences for qRT-PCR reactions were as follows: NORAD forward, 5′-TGATAGGATACATCTTGGACATGGA-3′ and reverse, 5′-AACCTAATGAACAAGTCCTGACATACA-3′; β-actin forward, 5′-AGCACAGAGCCTCGCCTT-3′ and reverse, 5′-CATCATCCATGGTGAGCTGG-3′; miR-30c-5p forward, 5′-AGCGTCGTATCCAGTGCAAT-3′ and reverse, 5′-GTCGTATCCAGTGCGTGTCG-3′; and U6 forward, 5′-CTCGCTTCGGCAGCACA-3′ and reverse, 5′-AACGCTTCACGAATTTGCGT-3′. Conditions were set as follows: 95°C for 30 s, 40 cycles at 95°C for 5 s, annealing, and extension at 60°C for 30 s, and melt curve analysis. U6 was used as an internal control for miR-30c-5p and human β-actin was used as an internal control for mRNA detection. The relative expression levels were calculated using the 2^−ΔΔCt^ method. Experiments were performed in triplicate.

### 2.6 Luciferase reporter assays

Human wild-type (WT)- or binding site mutant (MUT)-NORAD or LDHA 3′-UTR was amplified and inserted into the pSI-Check2 vector. HPMECs and A549 cells were seeded in 96-well plates and cultured for 24 h, followed by co-transfection with control miRNA or miR-30c-5p plus WT- or MUT-NORAD or LDHA 3′UTR using Lipofectamine 2000 (Invitrogen, Carlsbad, CA, USA). 48 h post transfection, firefly luciferase and *Renilla* luciferase activities were examined using the dual-luciferase reporter system (Luciferase Assay Reagent; Promega, San Luis Obispo, CA, USA). Experiments were performed in triplicate.

### 2.7 Measurements of LDH and Caspase-3 activity

The measurements of LDH and Caspase-3 activity were performed using the Lactate Dehydrogenase Activity Assay Kit (#MAK066, Sigma, Shanghai, China) and Caspase-3 Activity Assay Kit (Fluorometric) (#ab252897, Abcam, Cambridge, UK) according to the manufacturer’s instructions. Experiments were repeated three times.

### 2.8 RNA pull-down assay

The scramble control, sense, and antisense NORAD probes were biotin-labeled from RiboBio Co. Ltd (Guangzhou, China). Cell lysates were extracted using RIPA buffer (Sigma, Shanghai, China) and incubated with probes for 2 h. Streptavidin-coupled agarose beads (Thermo Fisher Scientific, Shanghai, China) were added into the mixture and incubated for 2 h. After washing, the miR-30c-5p was pulled down in the RNA complex. The amount of miR-30c-5p in the RNA complex was determined by qRT-PCR and Northern blot. Probes of sense and antisense NORAD are: Sense: 5′-GGUUGUAAACAGGAUGGCAUAGAGCUCUCUGCGCG-3′; Antisense: 5′-GGUGCAGAGAGCUCUAUGCCAUCCUGUUUACAGCG-3′.

### 2.9 Measurements of glucose metabolism

The glucose metabolism rate was evaluated by glucose consumption assay and the extracellular acidification rate (ECAR) assay using the Glucose Uptake Colorimetric Assay Kit (#MAK083) (Sigma-Aldrich, Shanghai, China) and the glycolysis stress kit (Agilent, Santa Clara, CA, USA) on a Seahorse XFp Analyzer (Agilent, Santa Clara, CA, USA) according to the manufacturer’s protocol. Results were normalized to the protein concentrations of each group. Experiments were repeated three times and performed in triplicate.

### 2.10 Cell viability assay

Viability of HPMECs under LPS treatment was determined by MTT (3-(4,5-dimethylthiazol-2-yl)-2,5-diphenyl-tetrazolium bromide) assay. Cells (1 × 10^5^ per well) were seeded in a 24-well plate for 24 h at regular cell culture condition. After LPS treatments, 0.1 mL of MTT solution (5 mg/mL in PBS) was added to cells followed by 2 h incubation. Lysis buffer containing dimethyl sulfoxide (DMSO) was added into each well and incubated for 4 h at 37°C. The optical density value was measured at 450 nm. Experiments were performed in triplicate and repeated three times.

### 2.11 Cell apoptosis assay

The cell apoptosis of HPMECs under LPS treatment was determined by flowcytometry using the Annexin V-FITC Apoptosis Kit (Thermo Fisher Inc., Waltham, MA, USA) according to the manufacturer’s instructions. In brief, HPMECs (3 × 10^5^ cells per well) were seeded into 6-well cell culture plates for 24 h. After LPS treatments, cells were collected by trypsinization and washed in suspension by cold PBS. Binding buffer (500 µL) was added into suspension and the Annexin-FITC and propidium iodide (PI) (5 µL of each) were added into each group and fully mixed. Cells were incubated for 15 min in the dark at room temperature. Analysis of cell apoptosis/death was performed using FACSCalibur flowcytometry (Becton Dickinson, San Jose, CA, USA). Experiments were repeated three times.

### 2.12 Western blotting

HPMECs were trypsinized and washed by PBS followed by incubation with lysis buffer and radioimmunoprecipitation buffer (Beyotime, Shanghai, China) containing protease inhibitor cocktail (Beyotime) on ice for 20 min. Protein concentration was determined using an Enhanced BCA Protein Assay Kit (Beyotime). Equal amount of protein sample (40 μg) was loaded into each well and resolved with 10% SDS-PAGE, followed by electro-transferring to a polyvinylidene fluoride (PVDF) membrane. Membranes were blocked with 5% BSA in PBST for 1 h at room temperature followed by incubation with primary antibodies (1:1,000) at 4°C overnight. The membranes were washed with TBST three times with 5 min gap between each wash. Membranes were incubated with secondary antibody for 1 h at room temperature. β-actin was a loading control. Protein expressions were detected using an ECL reagent (Santa Cruz Biotechnology). Experiments were repeated three times.

### 2.13 LPS-induced ALI experiments in *in vivo* rat model

This study was approved by the Institutional Animal Care and Use Committee of the Ruijin Hospital, Shanghai Jiao Tong University School of Medicine and in accordance with ARRIVE guidelines. All rats received regular care. Male Sprague-Dawley (SD) rats (400–450 g) were housed at 22–24°C of a 12:12 h light–dark cycle, with free access to food and water. Rats (*n* = 8 per group) were randomly assigned into seven groups: (1) sham, (2) LPS, (3) LPS + control shRNA, and (4) LPS + sh NORAD. Control shRNA or sh NORAD was transfected into rat lung tissues and 48 h after transfection, rats were anesthetized with 10% chloral hydrate (350 mg/kg, i.p.) and received i.p. injection of 0.9% saline solution (control) or LPS (5 mg/kg). Rats were euthanized 12 h after LPS treatment and the perfused lungs were harvested, and the lung pulmonary microvascular endothelial cells were isolated according to previous reports [18]. Cells were immediately stored in liquid nitrogen for downstream analysis.

### 2.14 Statistical analysis

Statistical analyses were performed using the Prism 7.0 software (GraphPad, San Diego, CA, USA). Data are expressed as the mean value ± standard deviation (mean value ± SD). Two-tailed Student’s *t*-test was applied for comparison of difference between two independent groups. One-way analysis of variance (ANOVA) followed by Holm-Sidak *post hoc* test was applied to determine significant differences among three or more groups. Experiments were conducted in triplicate. A value of *p* < 0.05 was considered to be statistically significant.

## 3 Results

### 3.1 NORAD is induced and involves in HPMECs death during LPS stimulation

**Figure 1:**
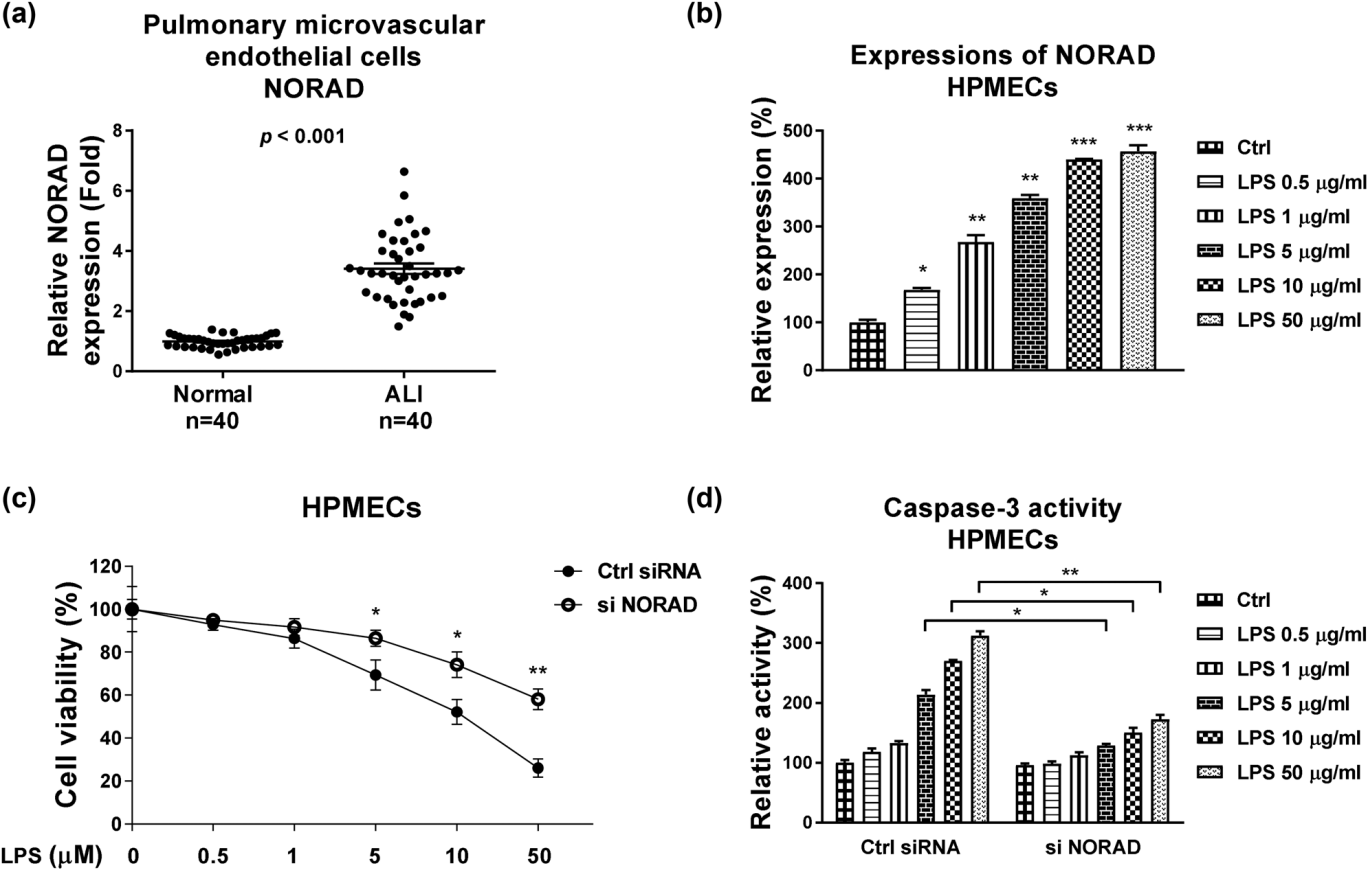
Roles of NORAD in the LPS-induced HPMEC cell injury. (a) Primary HPMECs from patient lung tissues (*n* = 40) and healthy controls were isolated and the expressions of NORAD were analyzed by qRT-PCR. (b) HPMECs were treated with LPS at 0, 0.5, 1, 5, 10, or 50 µg/mL. Expressions of NORAD were examined by qRT-PCR. (c) HPMECs were transfected with control siRNA or si NORAD, followed by treatment with LPS at the indicated concentrations. Cell responses were evaluated by MTT assay and (d) Caspase-3 activity assay. *, *p* < 0.05; **, *p* < 0.01; ***, *p* < 0.001.

To evaluate the roles of NORAD in the sepsis-evoked ALI, expressions of NORAD were compared in pulmonary microvascular endothelial cells from patients with ALI (*n* = 40) and healthy controls. Results from qRT-PCR showed that NORAD was significantly upregulated in pulmonary microvascular endothelial cells from ALI patients compared with those from controls (Figure 1a).

**Figure A1:**
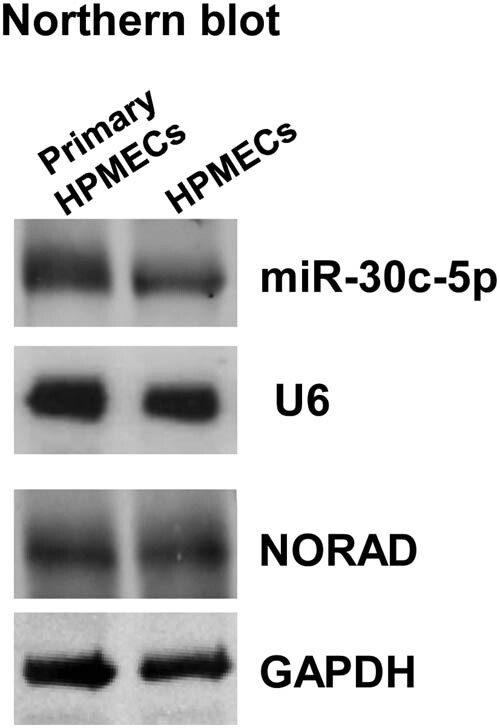
Northern blot results of NORAD and miR-30c-5p expressions. Isolated primary pulmonary microvascular endothelial cells and HPMECs cell line were subjected to Northern blot analysis. U6 and GAPDH were internal controls for miR-30c-5p and NORAD.

**Figure A2:**
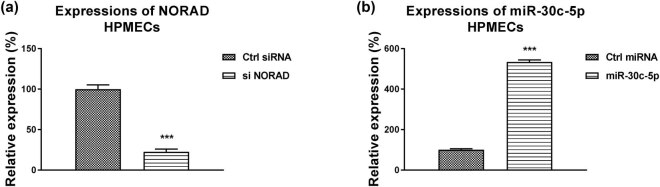
Transfection efficiency of si NORAD and miR-30c-5p in HPMECs. (a) HPMECs were transfected with control siRNA or si NORAD. Expressions of NORAD were examined by qRT-PCR. (b) HPMECs were transfected with control miRNA or miR-30c-5p. Expressions of miR-30c-5p were examined by qRT-PCR. ***, *p* < 0.001.

We then established an *in vitro* model of ALI through stimulation of HPMECs by LPS [19]. Expression of NORAD was significantly upregulated in LPS-treated HPMECs compared with control cells (Figure 1b). The qRT-PCR method for detecting NORAD and microRNA expressions were validated by Northern blot (Figure A1). To further investigate the biological functions of NORAD and the underlying mechanisms in the LPS-induced ALI/ARDS, NORAD was silenced in HPMECs (Figure A2a). NORAD knockdown without LPS stimulation did not affect cell viability. On the other way, HPMECs with low NORAD displayed attenuated cell death under LPS treatments compared with control siRNA transfected cells (Figure 1c). Expectedly, in control cells, LPS stimulation led to increased caspase-3 activity, which was antagonized by NORAD silencing in HPMECs (Figure 1d). Taken together, the above results suggest that NORAD plays important roles in the LPS-indued ALI and attenuation of NORAD could effectively protect the HPMECs injury.

### 3.2 LPS stimulation suppresses miR-30c-5p in HPMECs

**Figure 2:**
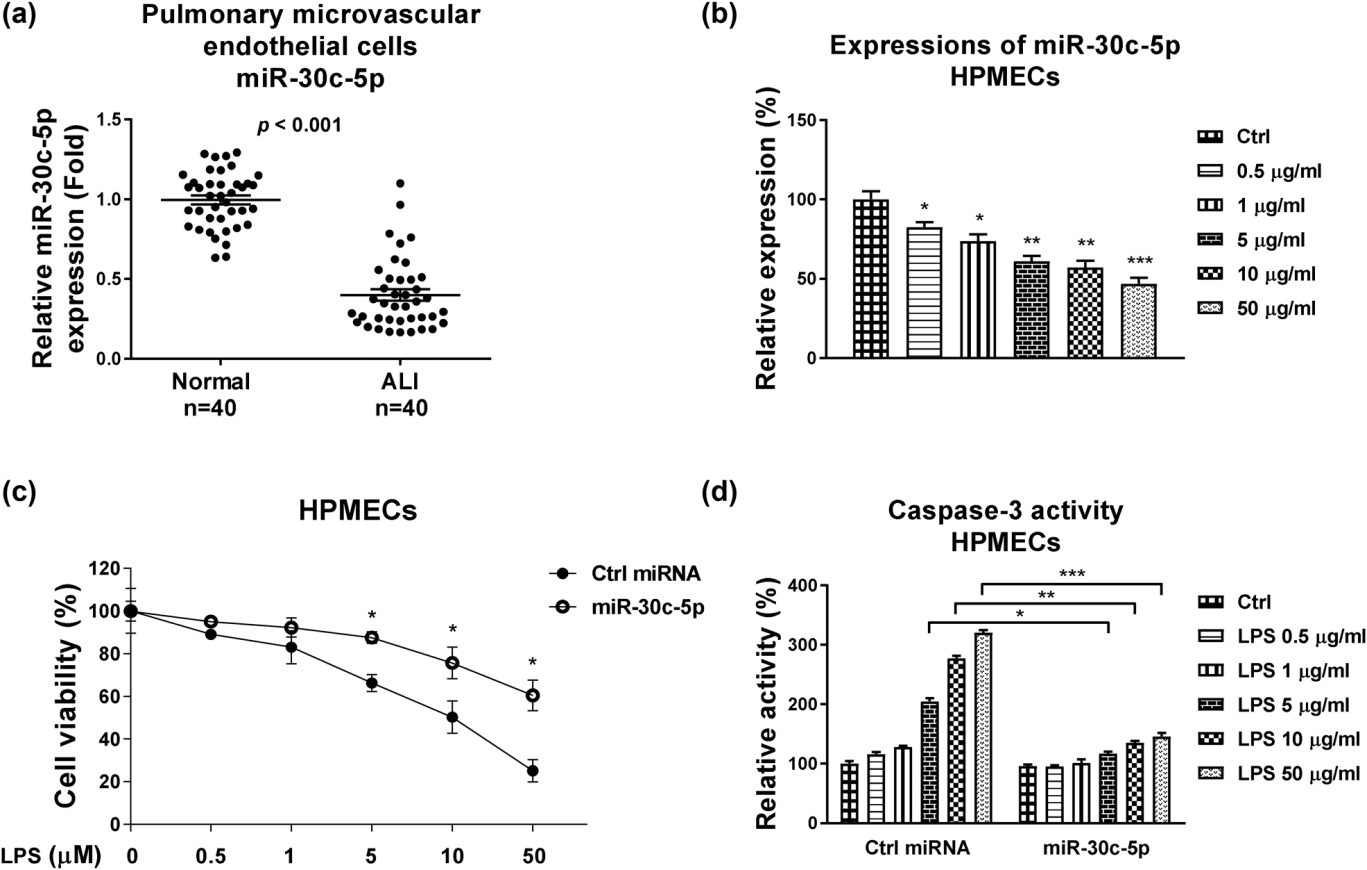
miR-30c-5p protects the LPS-induced apoptosis of HPMECs. (a) Primary HPMECs from patient lung tissues (*n* = 40) and healthy controls were isolated and the expressions of miR-30c-5p were analyzed by qRT-PCR. (b) HPMECs were treated with LPS at 0, 0.5, 1, 5, 10 or 50 µg/mL. Expressions of miR-30c-5p were examined by qRT-PCR. (c) HPMECs were transfected with control miRNA or miR-30c-5p, followed by treatment with LPS at the indicated concentrations. Cell responses were evaluated by MTT assay and (d) caspase-3 activity assay. *, *p* < 0.05; **, *p* < 0.01; ***, *p* < 0.001.

We then investigated the potential molecular mechanisms for the NORAD-protected HPMECs injury under LPS stimulation. Accumulating studies uncovered that lncRNAs could sponge miRNAs to form a ceRNA regulatory network which downregulates miRNA expressions, resulting in de-repression of miRNA target expressions [8]. Interestingly, we detected significantly downregulated miR-30c-5p in pulmonary microvascular endothelial cells from patients with ALI (*n* = 40) compared with healthy controls (Figure 2a). Consistently, miR-30c-5p expressions were remarkedly suppressed in LPS-treated HPMECs (Figure 2b), suggesting miR-30c-5p might act as an ALI-preventing role. Consequently, overexpression of miR-30c-5p (Figure A2b) effectively decreased the LPS-induced HPMECs death (Figure 2c) and caspase-3 activity (Figure 2d), suggesting an invert role of miR-30c-5p in the pathological processes of ALI.

### 3.3 NORAD downregulates miR-30c-5p by sponging it in HPMECs

**Figure 3:**
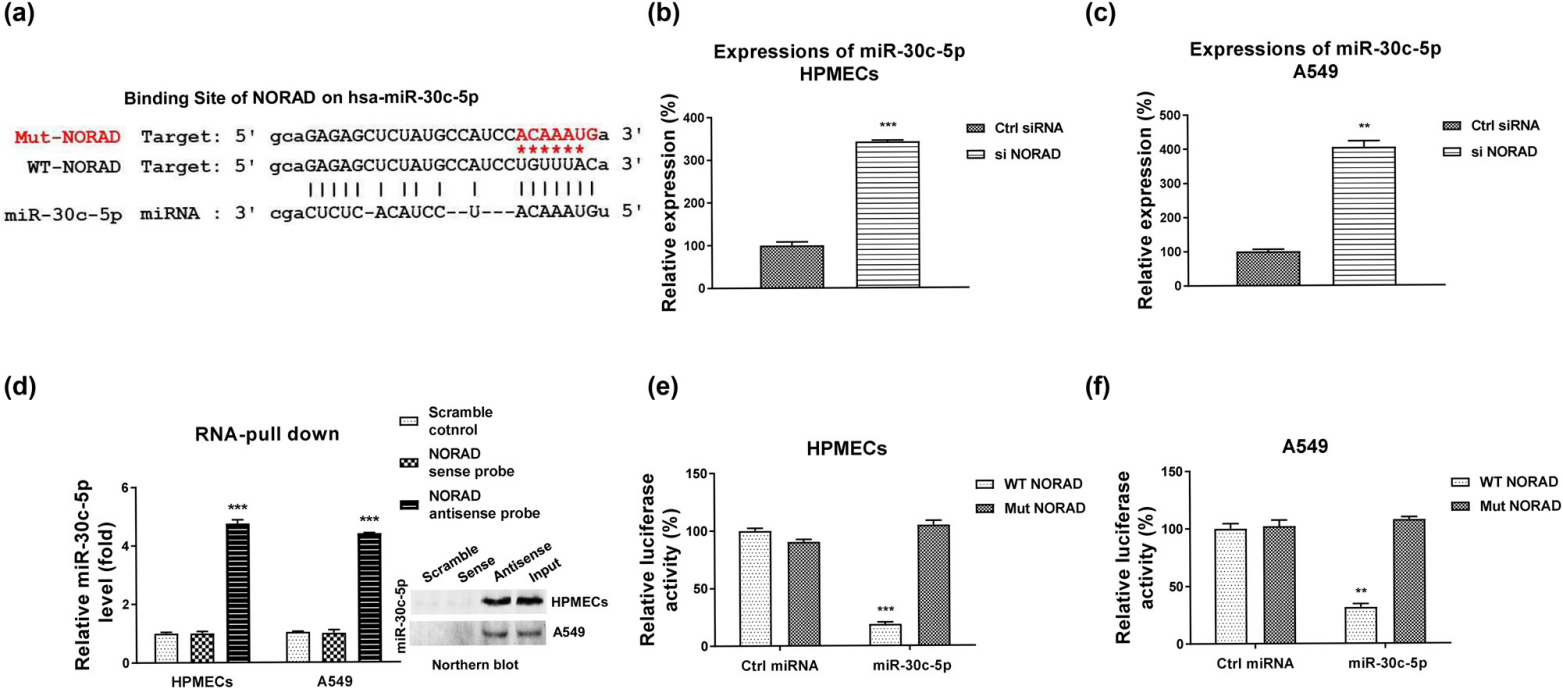
NORAD sponges miR-30c-5p in HPMECs. (a) Prediction of the binding of miR-30c-5p on NORAD from starBase. (b) HPMECs and (c) A549 cells were transfected with control siRNA or si NORAD. Expressions of miR-30c-5p were examined by qRT-PCR. (d) RNA pull-down assay was shown in HPMECs by incubation of biotin-labeled control, sense, or antisense NORAD probe with cell lysates. Enrichment of miR-30c-5p in the RNA complex was determined by qRT-PCR and Northern blot. (e) HPMECs and (f) A549 cells were transfected with control miRNA or miR-30c-5p with luciferase vector containing WT- or MUT-NORAD. Luciferase assay was performed. **, *p* < 0.01; ***, *p* < 0.001.

Bioinformatics analysis from the lncRNA service starBase2.0 illustrated that miRNA-30c-5p contains putative NORAD binding sites (Figure 3a). To assess whether NORAD could directly regulate miR-30c-5p expressions, HPMEC and A549 cells were transfected with control siRNA or NORAD siRNA. Expected results showed that cells with higher NORAD expressions displayed effectively attenuated miR-30c-5p expressions (Figure 3b and c). To verify the binding between lncRNA-NORAD and miR-30c-5p, RNA pull-down assay was performed. Biotin-labeled scramble, sense, or antisense NORAD probe was incubated with HPMEC and A549 cell lysate. Only biotin-labeled antisense NORAD probe could pull down enriched miR-30c-5p (Figure 3d). The endogenous miR-30c-5p was effectively pulled down by control and sense probe of NORAD (Figure 3d). Similar results were observed in A549 cells (Figure 3d). To validate whether NORAD could directly bind onto the seeding region of miR-30c-5p, HPMEC cells were co-transfected with control or miR-30c-5p or luciferase vector with insertion of WT- or binding site MUT-NORAD. As we expected, luciferase activity of HPMEC cells with WT-NORAD and miR-30c-5p co-transfection was significantly suppressed (Figure 3e and f). Meanwhile, no significant change was detected in the luciferase activity of cells which were co-transfected with miR-30c-5p or control miRNA plus MUT-NORAD (Figure 3e and f). Taken together, these results demonstrated that NORAD blocked miR-30c-5p expressions via sponging it.

### 3.4 Glycolysis of HPMEC was activated by LPS to induce cell apoptosis

**Figure 4:**
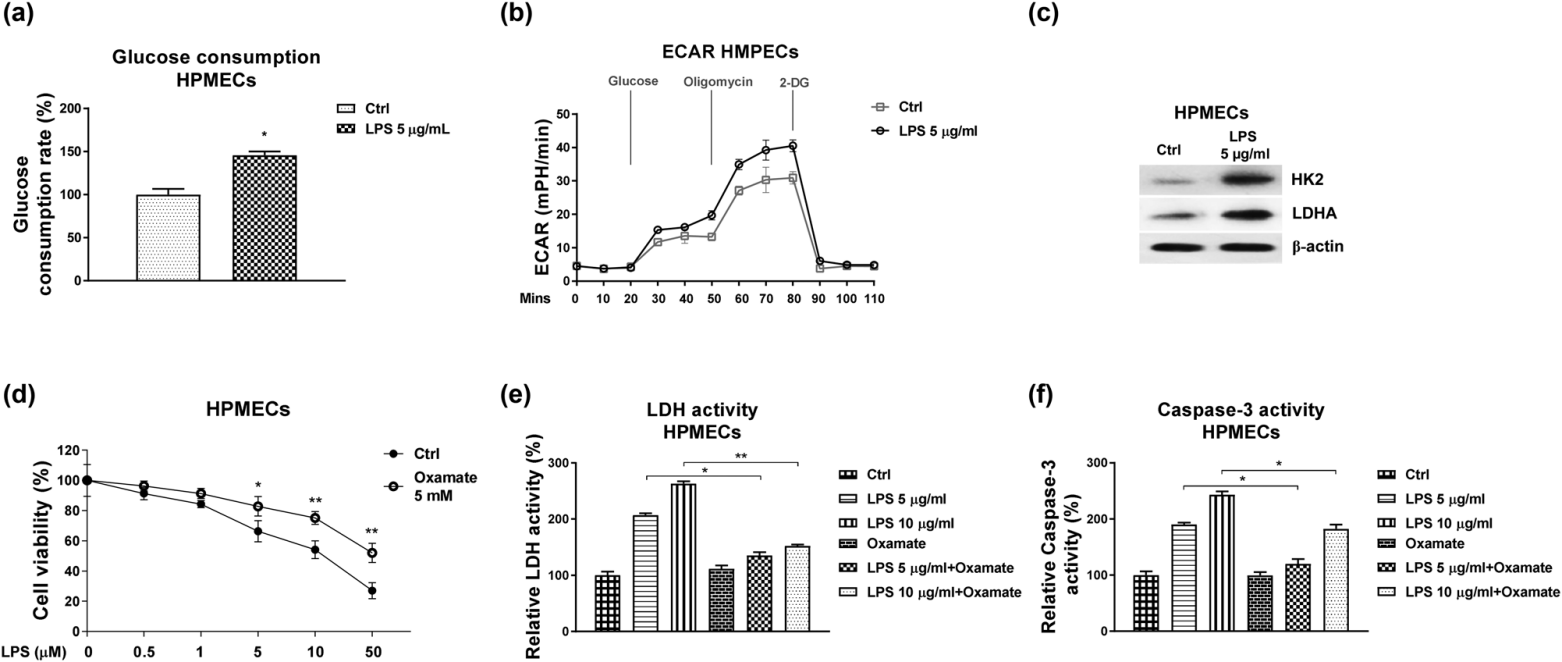
Glycolysis rate of HPMECs is elevated by LPS stimulation. (a) HPMECs were treated with control or LPS at 5 µg/mL. The glucose consumption, (b) ECAR and (c) glycolysis enzymes were examined. (d) HPMECs were treated with control or Oxamate at 5 mM, followed by treatments with LPS at the indicated concentrations. Cell viability was examined by MTT assay. (e and f) HPMECs were treated with LPS alone or with Oxamate at the indicated concentrations. The LDH activity and Caspase-3 activity were examined. *, *p* < 0.05; **, *p* < 0.01.

We then investigated the cellular mechanisms for the LPS-induced cell injury of HPMECs. Previous studies reported that in an LPS-induced acute lung injury mouse model, the cellular glucose metabolism of HPMECs was significantly elevated, leading to lactate secretion [20], suggesting targeting the glycolysis might contribute to protecting the LPS-induced HPMECs injury. We then evaluated the glycolysis rate of HPMECs under LPS treatments. Results in Figure 4a and b showed that HPMECs with LPS treatment displayed significantly increased glucose uptake and ECAR. Moreover, the glycolysis key enzymes, HK2, and LDHA were significantly upregulated under LPS treatments (Figure 4c). To verify whether LPS induced HPMECs injury through activating the glycolysis, the HPMECs were pre-treated with glycolysis inhibitor, Oxamate for 48 h, followed by LPS stimulation. Expectedly, blocking glycolysis effectively prevented the cell apoptosis induced by LPS (Figure 4d). Consistently, the LDH release and activity of caspase-3 were inhibited with glycolysis inhibitor under LPS treatments (Figure 4e and f). In summary, our results suggest that blocking the LPS-induced glycolysis might be an effective approach to protect ALI.

### 3.5 Reverse effects of NORAD and miR-30c-5p in modulating anaerobic glycolysis

**Figure 5:**
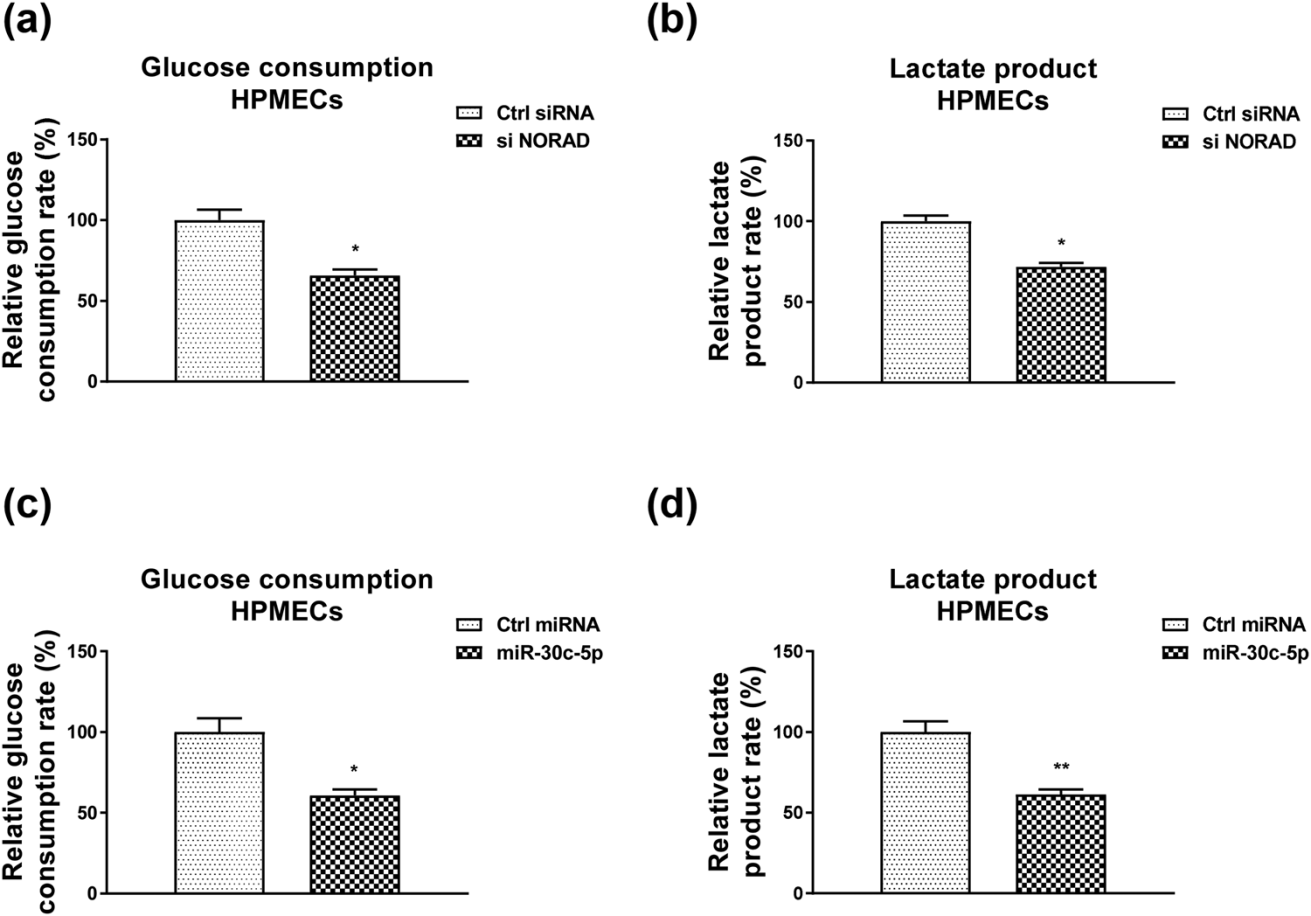
Invert roles of NORAD and miR-30c-5p in glycolysis. (a) The glucose consumption and (b) ECAR were examined in HPMECs without or with NORAD silencing. (c) The glucose consumption and (d) ECAR were examined in HPMECs without or with miR-30c-5p overexpression. *, *p* < 0.05; **, *p* < 0.01.

The above results revealed reverse roles of NORAD and miR-30c-5p in the LPS-induced HPMECs injury. To investigate whether NORAD and miR-30c-5p modulate glycolysis in an invert pattern, effects of NORAD silencing and miR-30c-5p overexpression on glucose metabolism were assessed. Results in Figure 5a demonstrated that silencing NORAD significantly suppressed glucose uptake. In addition, the lactate product was inhibited in NORAD siRNA transfected cells (Figure 5b). Consistently, overexpression of miR-30c-5p remarkedly blocked the glycolysis rate of HPMECs (Figure 5c and d).

### 3.6 miR-30c-5p targets LDHA to attenuate glycolysis of HPMECs

**Figure 6:**
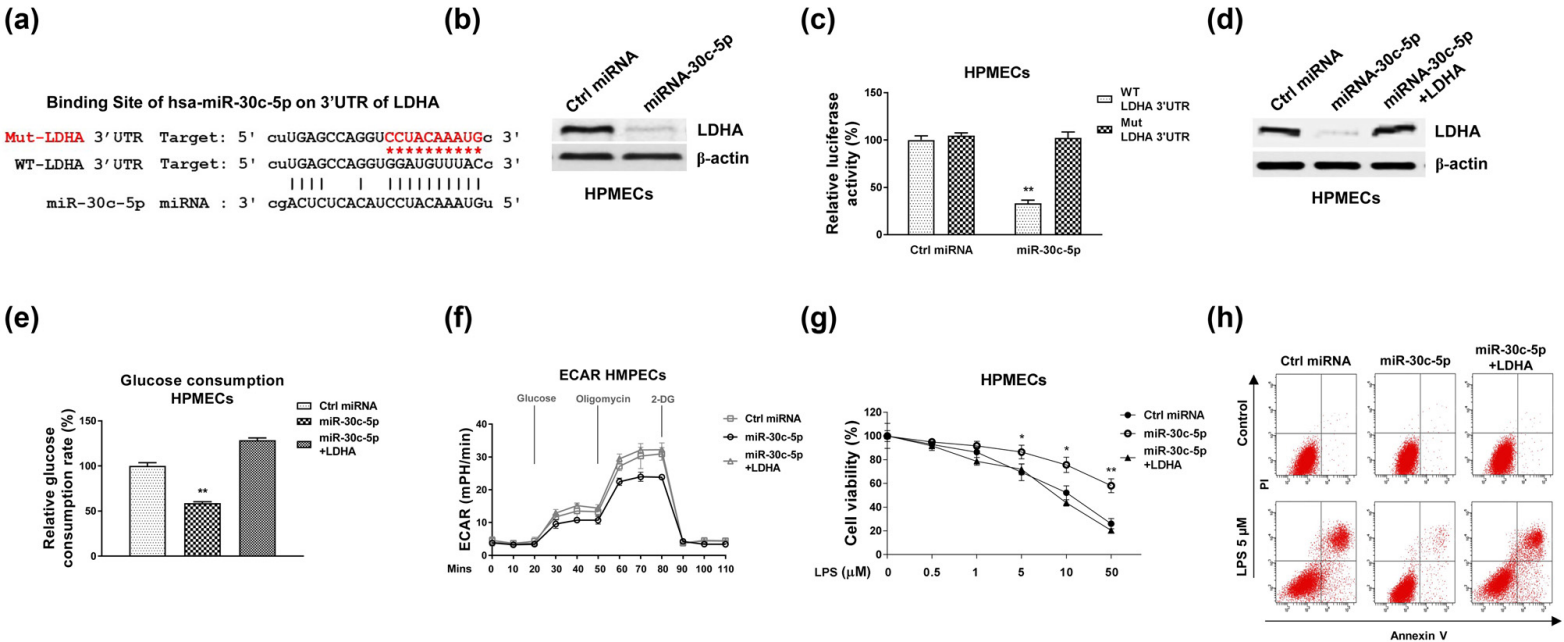
miR-30c-5p directly targets LDHA in HPMECs. (a) Prediction of the binding of miR-30c-5p on 3′UTR of LDHA from starBase. (b) HPMECs were transfected with control miRNA or miR-30c-5p. Expressions of LDHA were examined by western blot. (c) HPMECs were transfected with control miRNA or miR-30c-5p with luciferase vector containing WT- or MUT-LDHA 3′UTR. Luciferase assay was performed. (d) HPMECs were transfected with control miRNA, miR-30c-5p alone, or with LDHA. Expressions of LDHA were examined by western blot. (e) The glucose consumption and (f) ECAR were examined in the above transfected cells. (g) The transfected cells were treated with LPS at the indicated concentrations. Cell survival rates were determined by MTT assay and (h) Annexin V apoptosis assay. *, *p* < 0.05; **, *p* < 0.01.

**Figure A3:**
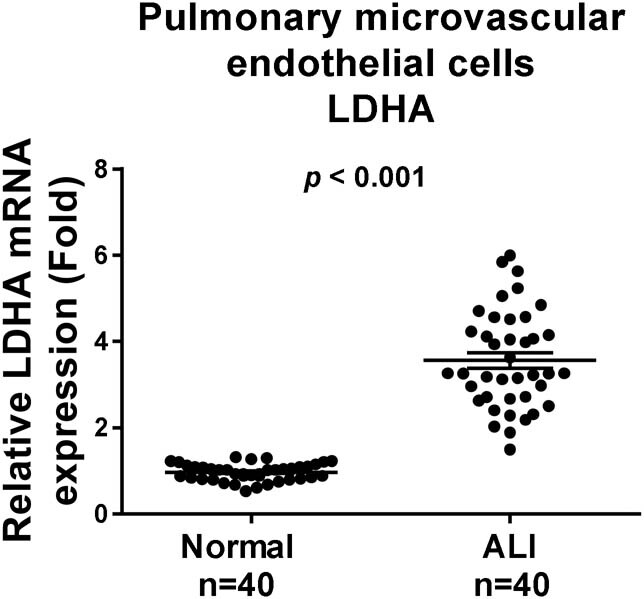
Expression of LHDA in ALI. Primary human pulmonary microvascular endothelial cells from patient lung tissues (*n* = 40) and healthy controls were isolated and the expressions of LDHA were analyzed by qRT-PCR.

Accumulating studies described miRNAs function through binding to the 3′UTR of their target mRNAs to interfere the mRNA stability [21]. We thus investigated the targets of miR-30c-5p in the LPS-induced lung endothelial injury. Targets of miR-30c-5p were predicted from starBase2.0 non-coding RNA service. We observed LDHA (Figure 6a), which is a glycolysis key enzyme that converts the pyruvate to lactate, is a potential target of miR-30c-5p. Consistent results showed that LDHA was significantly upregulated in pulmonary microvascular endothelial cells from patients with ALI (*n* = 40) compared with healthy controls (Figure A3), suggesting LDHA is positively associated with ALI. Contrarily, we detected that overexpression of miR-30c-5p significantly downregulated LDHA protein expression in HPMECs (Figure 6b). To obtain the direct evidence of miR-30c-5p–LDHA interaction, luciferase reporter assays were performed. HPMEC cells were co-transfected with control or miR-30c-5p or luciferase vector with insertion of WT or binding site MUT 3′UTR of LDHA. Expectedly, luciferase activity of HPMEC cells with WT-LDHA 3′UTR and miR-30c-5p co-transfection was significantly suppressed but no significant change was detected in the luciferase activity of cells which were co-transfected with miR-30c-5p or control miRNA plus Mut-LDHA 3′UTR (Figure 6c).

We then asked whether the miR-30c-5p-mediated HPMECs protection was through targeting LDHA. Rescue experiments were performed by transfection of control miRNA, miR-30c-5p alone, or plus LDHA overexpression vector into HPMEC cells. Results from western blot showed the combined transfection of miR-30c-5p with LDHA successfully recovered the LDHA protein expression (Figure 6d). Furthermore, rescue of LDHA in miR-30c-5p-overexpressing HPMEC cells effectively restored the glucose uptake (Figure 6e) and ECAR (Figure 6f). Importantly, HPMEC cells with recovery of LDHA exhibited obviously increased sensitization to LPS by cell viability assay (Figure 6g) and apoptosis assay (Figure 6h). Taken together, the above data consistently demonstrated that miR-30c-5p protects HPMECs under LPS-induced cell injury via direct targeting of LDHA-glycolysis pathway.

### 3.7 Blocking NORAD protects LPS-induced cell death through the miR-30c-5p-LDHA axis

**Figure 7:**
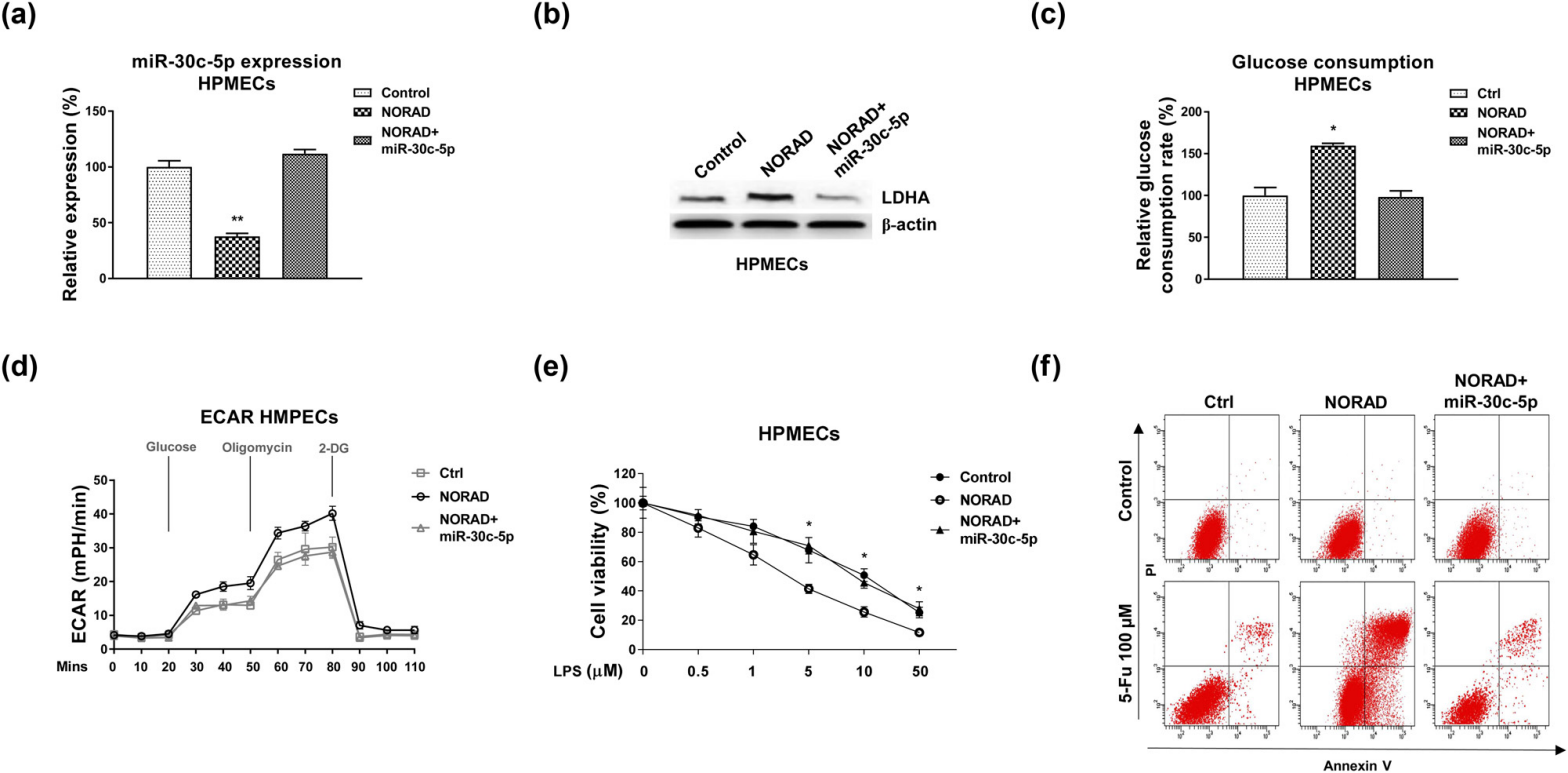
Roles of the NORAD-miR-30c-5p-LDHA axis in the LPS-induced HPMECs dysfunction. (a) HPMECs were transfected with control, NORAD alone, or with miR-30c-5p. Expressions of miR-30c-5p and (b) LDHA were examined by qRT-PCR and western blot, respectively. (c) The glucose consumption and (d) ECAR were examined in the above transfected cells. (e) HPMECs were transfected with control, NORAD, NORAD plus miR-30c-5p were treated with control or LPS. Cell survival rates were determined by MTT assay and (f) Annexin V apoptosis assay. *, *p* < 0.05; **, *p* < 0.01.

Finally, we investigated whether the NORAD-modulated HPMECs protection under LPS treatment was through the miR-30c-5p-LDHA axis. Mechanism rescue experiments were conducted by co-transfection of control vector, NORAD overexpression vector alone, or plus miR-30c-5p into HPMECs. Western blot results showed overexpression of NORAD significantly suppressed miR-30c-5p (Figure 7a) and upregulated LDHA protein expressions (Figure 7b). Recovery of miR-30c-5p effectively re-suppressed LDHA expression (Figure 7a and b). Consistently, restoration of miR-30c-5p in NORAD-overexpressing HPMECs successfully recovered the glucose consumption (Figure 7c) and ECAR (Figure 7d) compared with NORAD transfection alone. Moreover, restoration of miR-30c-5p in NORAD-overexpressing HPMECs effectively protected cells under the LPS-induced apoptosis by cell viability assay (Figure 7e) and Annexin V apoptosis assay (Figure 7f).

**Figure 8:**
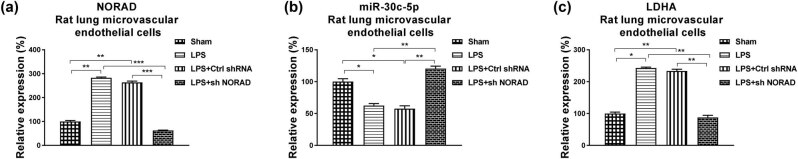
Roles of NORAD-miR-30c-5p-LDHA axis in an ALI rat model. (a) Control or LPS was injected i.p. to induced ALI in rats 48 h after transfection of control shRNA or sh NORAD. Rat lung endothelial cells were isolated. Expressions of NORAD, (b) miR-30c-5p and (c) LDHA mRNA were examined. *, *p* < 0.05; **, *p* < 0.01; ***, *p* < 0.001.

**Figure 9:**
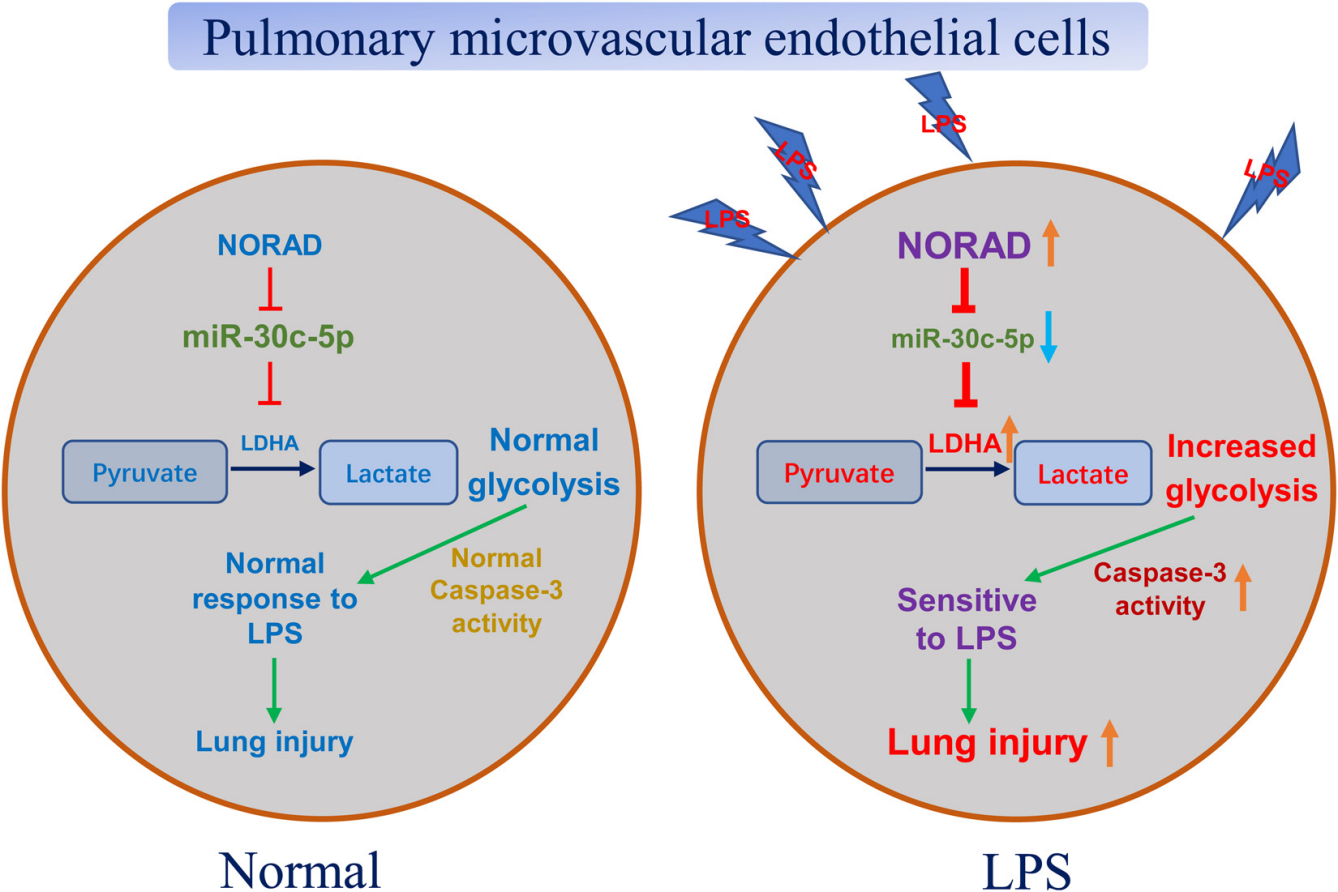
A working model for the NORAD-miR-30c-5p-LDHA axis in the LPS-induced HPMECs dysfunction.

To further validate the above *in vitro* conclusions, we performed *in vivo* experiments by establishing an ALI rat model. Control or LPS was injected i.p. to induce ALI in rats 48 h after transfection of control shRNA or sh NORAD. Rat lung endothelial cells were isolated. Consistent with *in vitro* results, rat lung endothelial cells in LPS treatment group exhibited increased NORAD and LDHA expression and decreased miR-30c-5p expression (Figure 8a–c). Furthermore, silencing NORAD effectively recovered miR-30c-5p expression and downregulated LDHA expression in rat lung microvascular endothelial cells under LPS treatments (Figure 8a–c). Generally, these *in vitro* and *in vivo* results consistently validated that the NORAD-promoted HPMECs cell death under LPS through modulating the miR-30c-5p-LDHA axis (Figure 9).

## 4 Discussion

ALI is a devastating human malignancy which is characterized by excessively uncontrolled inflammation, lung epithelial and endothelial cell dysfunction, and oxidative damage [1]. Thus, it is urgent to investigate effectively therapeutic approaches for ALI treatment. Accumulating evidence revealed that pulmonary endothelium plays essential roles which contribute to the development and progression of ALI [2]. The LPS-stimulated HPMECs has been widely used as an *in vitro* ALI model to study the effects and molecular mechanisms of lung endothelial cell dysfunction [6]. NORAD has been reported to be highly expressed in lung tissues and is associated with tumorigenesis and progresses of lung cancer [14,22]. In this study, we reported a lncRNA NORAD-regulated endothelial cell dysfunction under LPS stimulation using an *in vitro* HPMECs model and *in vivo* rat LPS model. NORAD was observed to be significantly induced under LPS treatment. Expectedly, silencing NORAD effectively protected HPMECs against the LPS-induced HPMECs apoptosis. These results highlighted that NORAD is a potentially therapeutic target in management of ALI.

Recent studies uncovered that lncRNAs function as sponges of miRNAs to suppress their expressions, leading to re-activation of downstream target mRNAs [8]. To explore the molecular targets of NORAD, we identified miR-30c-5p as a direct target of NORAD which significantly downregulated miR-30c-5p expression in HPMECs. RNA pull-down assay and luciferase assay consistently demonstrated that NORAD was associated with miR-30c-5p to form a ceRNA regulatory network in lung endothelial cells. miR-30c-5p is a member of miR-30 family which is conservely expressed in lung tissues [23]. In this study, we detected miR-30c-5p was suppressed during the LPS-stimulated lung endothelial cell death, suggesting miR-30c-5p plays a protective role in ALI.

Accumulating studies revealed that a series of pathophysiological alterations such as endothelial cell glucose metabolism are associated with ALI-induced lung sepsis [14]. During ALI, hyperlactatemia occurs due to the inadequate oxygen supply-activated upregulation of anaerobic glycolysis [24]. Anaerobic glycolysis is the metabolization of glucose to lactate controlled by multiple speed-limiting glycolytic enzymes such as LDHA [25]. A recent study showed blocking glycolysis pathway by inhibitor, PFKFB3, 3-(3-pyridinyl)-1-(4-pyridinyl)-2-propen-1-one(3PO), which led to decreased glucose uptake and suppression of glycolytic flux to lactate, was an effective approach to prevent the sepsis-related ALI [24], indicating inhibition of cellular glycolysis is a potential way to treat ALI. In this study, LPS exposure remarkedly stimulated the glycolysis rate of HPMECs, the glucose consumption, and ECAR as well as the glycolysis enzymes were significantly promoted. We then asked whether the NORAD-miR-30c-5p axis-modulated lung endothelial cells dysfunction was through targeting the LDHA-glycolysis pathway. Rescue experiments showed that recovery of miR-30c-5p in NORAD-overexpressing HPMECs cells effectively overrode the NORAD-promoted glucose metabolism and protected the LPS-induced lung endothelial cell death.

In conclusion, the current study demonstrated that inhibition of NORAD effectively ameliorated lung endothelial cell damage by LPS stimulation through modulating the miR-30c-5p-LDHA-anearobic glycolysis pathway.
